# Predictors of cannabis and tobacco co-use in youth: exploring the mediating role of age at first use in the population assessment of tobacco health (PATH) study

**DOI:** 10.1186/s42238-021-00072-2

**Published:** 2021-06-01

**Authors:** Crystal Lederhos Smith, Brittany Rhoades Cooper, Andre Miguel, Laura Hill, John Roll, Sterling McPherson

**Affiliations:** 1 Elson S. Floyd College of Medicine, Washington State University, P.O. Box 1495, Spokane, WA 99210-1495 USA; 2 Human Development, Washington State University, Spokane, WA USA

**Keywords:** Co-use, Cannabis, Tobacco, Adolescent, Age at first use, Sensation-seeking, Alcohol

## Abstract

**Background:**

Adolescents often use substances such as tobacco and cannabis. Co-use of these substances can lead to physical, mental, and psychosocial difficulties beyond that which would be anticipated by simple additivity of their individual effects.

**Methods:**

We aimed to examine the mediating role of age at first use of cannabis or tobacco (AU) between youth factors of internalizing, externalizing, and sensation seeking and two co-use outcomes (lifetime; last 30 days). Path analytic modeling using data from youth age 12–17 who had tried cannabis or tobacco at least once in their lives and participated in the Population Assessment of Tobacco Health (PATH) waves one and two (collected 2013–2015; n=3,847; approximately 46% female) study allowed us to examine these relationships.

**Results:**

The lifetime use model indicated significant direct (internalizing (*B* = 0.18), externalizing (*B* = 0.30), sensation seeking (*B* = 0.15)) and indirect relationships (internalizing (*B* = 0.18), externalizing (*B* = 0.33), sensation seeking (*B* = 0.10)) between each of the three youth factors, the mediator (AU) and the lifetime co-use outcome (*p* < 0.05 for all). A direct relationship between AU and lifetime co-use was also observed (*B* = − 1.54). In the past 30-day use model, significant direct paths from AU (*B* = − 0.49) and sensation seeking (*B* = 0.06) to past 30-day use were present (*p* < 0.05 for all).

**Discussion:**

Examination of mediation by AU in the relationships between youth factors and youth co-use of cannabis and tobacco is an important step in understanding these complex relationships. This study is strengthened by the use of a large, nationally representative sample, yet is limited by several factors, such as the use of a secondary dataset and the use of youth self-report.

**Conclusions:**

Based on the findings, programs or interventions targeting youth factors of internalizing, externalizing, and sensation seeking as well as interventions aiming to stave off AU should promote decreased tobacco and cannabis co-use. Sensation seeking and AU appear to be the most influential factors and should be considered when developing and promoting prevention policies/programs for higher risk youth populations.

## Background

Cannabis and tobacco (smoked and smokeless) are two of the most commonly used substances among youth in the United States (US), with co-use of these substances being frequent in this population (5.4%), and even more common than exclusive use of cannabis (3.9%) or of tobacco (2.2%) (Schauer and Peters 2018). Co-use of these two substances has been defined in several manners, including concurrent use, within the same time frame (such as the last 30 days); combined use, use of the two substances mixed together (e.g., blunts or spliffs); and simultaneous use (e.g., a cannabis joint and a tobacco cigarette used one after the other). For the purposes of this manuscript, co-use is defined as concurrent use. This substance use overlap is concerning as users of both tobacco and cannabis are at higher risk for developing respiratory distress, reduced lung functioning (Strong et al. 2018; Taylor et al. 2002), nicotine use disorder (Agrawal et al. 2004; Wang et al. 2016), psychosocial problems (Peters et al. 2012), and some forms of cancer (Lacson et al. 2012) compared to users of either substance alone. Furthermore, the co-use of these two substances have reciprocal and graduating effects, with each substance promoting a higher likelihood of initial use of the other substance, and with concurrent use leading to a steeper escalation in the use of both substances (Agrawal et al. 2012; Agrawal and Lynskey 2009; Agrawal et al. 2011; Badiani et al. 2015; Ream et al. 2008). It is important to not only identify factors that impact the use and co-use of these substances, but also to examine factors that potentially mediate these effects, such as the age at which an adolescent first uses the substances.

Strong empirical evidence supports our examination of these specific youth risk factors. Some of the strongest evidence shows that early (prior to age 17) age at first use of tobacco and cannabis is a pivotal factor consistently related to future quantity and number of substances used and influences long-term health outcomes (Agrawal et al. 2012; Kaplan et al. 1986; McPherson et al. 2014; Substance Abuse and Mental Health Services Administration (SAMHSA) 2014). For example, studies indicate that younger age at first use of substances is associated with continued substance use and heavy use of substances, use of subsequently more harmful drug classes, and poor health outcomes, such as respiratory distress and reduced lung functioning, later in life (Agrawal et al. 2006; DuRant et al. 1999; Grant 1998; Gruber et al. 2012). Early age of first use of cannabis, specifically, is related to increased likelihood of future use and decreased cognitive function (Gruber et al. 2012), as well as increased engagement in health risk behaviors (DuRant et al. 1999), while early age of smoking initiation has been linked to a cluster of risky behaviors in youth (e.g., use of other substances and fighting) (DuRant et al. 1999; Grant 1998).

Youth internalizing disorders and related behaviors, such as anxiety and depression, have also been linked to both cannabis and tobacco use (Conway et al. 2018; Leventhal et al. 2016; Marmorstein et al. 2010; Tercyak and Audrain 2002). There is also evidence for increased cannabis use in youth exhibiting externalizing behaviors and disorders (e.g. impulsivity, delinquency, disruptive behavior, conduct disorder, oppositional defiant disorder, attention-deficit/hyper-activity disorder) (Armstrong and Costello 2002; Brinkman et al. 2015; Groenman et al. 2013; Gruber et al. 2014; Haas et al. 2018; Kosterman et al. 2000; Lee et al. 2011). Sensation seeking behaviors (e.g., liking frightening and exciting experiences and friends (Hoyle et al. 2002) have also been linked to earlier age of first use of substances, as well as increased risk for polysubstance use (Agrawal et al. 2012; Ball et al. 1994).

There is limited research specifically examining risk factors for cannabis and tobacco co-use in comparison to single use. However, some research suggests different risk factors for polysubstance use (use of three or more substances) than single substance use. For example, polysubstance users are more likely to have aggression, impulsivity, and sensation seeking tendencies (Ball et al. 1994; Chen and Jacobson 2012; Martinotti et al. 2009), whereas single substance users are more likely to have experienced depressive symptoms (Martinotti et al. 2009). Additional adolescent research indicates specifically that there is an increased risk for tobacco and cannabis co-use for those youth exhibiting sensation seeking and externalizing behaviors (Agrawal et al. 2012; Agrawal et al. 2006).

The Social Development Model (SDM) suggests that factors that reduce risk of negative outcomes (i.e., protective factors) do so by moderating or mediating the relationship between risk factors and problem behaviors (Catalano and Hawkins 1996; Catalano and Hawkins 1996; Hawkins 1996). When applied to the examination of tobacco and cannabis use in youth, the SDM allows us to account for the multiple risk factors of interest, including internalizing, externalizing, sensation seeking, and the age at which youth first use tobacco or cannabis, and to explore the mediating mechanism through which these risk factors are associated with substance use outcomes. The SDM is useful in this context to parse out whether the mechanism through which youth factors are influencing co-use is their impact on age at first use. In addition, the SDM has been validated among samples of youth who use substances (Catalano and Hawkins 1996; Lonczak et al. 2001; Nuño et al. 2019), making it an appropriate justification for the mediation model that is tested in the analyses, however, although the SDM and literature has explored substance use, little research is published examining co-use of tobacco and cannabis as compared to single substance use.

In the current study, we examine the specific combination of tobacco (cigarette, dissolvable, filtered cigar, traditional cigar, smokeless tobacco, snus, hookah, pipe tobacco, cigarillo, bidi, kretek, and e-cig use) and cannabis use, compared to their use individually, in an effort to determine whether there are differences in the relationships between youth factors and single-use versus co-use outcomes. We tested a model for tobacco and cannabis, focusing on youth factors as predictors. Specifically, based on existing theory and empirical evidence, we examined age at first use of tobacco or cannabis as a mediator of the relationships between the youth factors of internalizing, externalizing, and sensation seeking, and tobacco and cannabis use outcomes. Indeed, alcohol use research by Hawkins et al. (1997) indicates that age at first use of alcohol fully mediates the relationships between several demographic characteristics and family environment on alcohol misuse outcomes. We utilized a similar model to examine the potential mechanism of action through which youth factors exert their impact on co-use. We hypothesized that age at first use of tobacco or cannabis would partially mediate the relationships found between the youth factors of internalizing, externalizing, and sensation seeking and tobacco and cannabis use and co-use outcomes 1 year later.

## Methods

### Data

Data for this study come from waves one, collected in 2013–2014, and two, collected in 2014–2015, of the Population Assessment of Tobacco Health (PATH), an ongoing, longitudinal cohort study. Youth participants completed interviews using Audio Computer-Assisted Self-Interviewing. For a full description of the data collection, study design, and methodology, see Hyland et al. (2016).

Young adults, African American adults, and tobacco users were oversampled for the PATH study. Due to this oversampling, non-response, and the use of a four-stage sample approach, all estimates in this study have been calculated using a weighting procedure, unless otherwise noted. The specific weighting procedure and explanations can be found in the PATH Study Public-Use Files (PUF) User Guide located at https://www.icpsr.umich.edu/icpsrweb/NAHDAP/series/606.

### Participants

Data for this study, come from the PATH dataset, are a nationally representative sample of youth ages 12–17 years (n=13,651). From this group we used all 12–17 year old’s who had tried cannabis or tobacco at some point in their life (n=3857) and who completed measures in wave one and two of the PATH study. Our sample was approximately 46% female; 50% 12–14 years old, and 50% 15–17 years old). No other inclusion criteria were required. Participants for this study were identified using a stratified, address-based, four-stage, area-probability sampling approach. This secondary data analysis was determined to be exempt by the Washington State University (WSU) Institutional Review Board (IRB).

### Measures

#### Background characteristics

Measures of background characteristics were collected through youth self-report. Youth provided information on their grade level, gender, Hispanic origin, and race. Each of these factors was included in the models as covariates.

#### Youth factors

Youth measures of internalizing and externalizing problems included the modified Global Appraisal of Individual Needs-Short Screener (GAIN-SS) (Dennis et al. 2008) and for sensation seeking, the modified Brief Sensation Seeking Scale (Hoyle et al. 2002), both established as reliable and valid measures.

#### Age at first use

The age at first use variable was generated by using the age category at which a youth first used either tobacco or cannabis, whichever happened earlier. Age at first use of tobacco was evaluated using questions of the format “When you first tried cigarette smoking, even one or two puffs, were you…” with response options of (1) less than 12 years old, (2) 12–14 years old, and (3) 15–17 years old. Questions in this format were asked for cigarette, dissolvable, filtered cigar, traditional cigar, smokeless tobacco, snus, hookah, pipe tobacco, cigarillo, bidi, kretek, and e-cig use. The final age at first use of tobacco variable was created by using the earliest age at first use of any of these substances. In order to measure age at first use of cannabis, we used the item “When you first used marijuana, hash, THC, grass, pot, or weed, were you…” with response options of (1) less than 12 years old, (2) 12–14 years old, and (3) 15–17 years old. Data for age at first use were taken from wave two, in an effort to capture first time use that occurred after wave one measurements.

#### Cannabis and tobacco use and co-use

Cannabis use was measured using the questions, “Have you ever used marijuana, hash, THC, grass, pot, or weed?” (yes or no) and “How long has it been since you last used marijuana, hash, THC, grass, pot or weed? Was it …” (1) within the past 30 days; (2) more than 30 days ago, but within the past year; or (3) more than a year ago. We recoded the second question into a dichotomous variable indicating whether the youth had used in the past 30 days or not. Information on tobacco use is available in dichotomous variables indicating whether the respondents had ever (yes or no) or in the past 30 days (yes or no) used tobacco. Tobacco use is based on an extensively detailed set of individual questions asked about the use of various types of tobacco products (e.g., cigarette, dissolvable tobacco, cigar, kretek, bidi, hookah).

We derived two binary variables, past 30-day use status and lifetime use status, from the questions above. Each of the variables has the following categories: (0) single substance use (just tobacco or just cannabis), (1) co-use (use of both tobacco and cannabis).

For examples of questions and response options for family and youth domains, please see Table 1.

**Table 1:** Sample survey items and response options comprising psychological constructs of sensation-seeking, internalizing, and externalizing

Construct (# of items per construct)	Sample items	Response options
Sensation seeking (3)	Please tell me how much you agree or disagree with each of the following statements: - I like to do frightening things.	1) Strongly agree, 2) Agree, 3) Neither agree nor disagree, 4) Disagree, 5) Strongly disagree
	- I like new and exciting experiences, even if I have to break the rules.	
Internalizing (4)	When was the last time that you had significant problems with… - Feeling very anxious, nervous, tense, scared, panicked, or like something bad was going to happen?	1) Past month, 2) 2–12 months ago, 3) Over a year ago, and 4) Never
	- Feeling very trapped, lonely, sad, blue, depressed, or hopeless about the future?	
Externalizing (6)	When was the last time you did the following two or more times … -Started physical fights with other people? - Gave answers before the other person finished asking the question?	1) Past month, 2) 2–12 months ago, 3) Over a year ago, and 4) Never

Items are from the Population Assessment of Tobacco Health (PATH) study

### Analysis

Data organization and cleaning were conducted in STATA (StataCorp 2011) and analyses were conducted in Mplus 8 (Muthen and Muthen 1998-2010). Missing data was managed using maximum likelihood (MLR), according to the most current recommendations (Enders 2001; McPherson et al. 2013). All exogenous covariates were from wave one data and outcomes from wave two data, collected 1 year later. Because grade and age were highly correlated with each other in our sample of adolescents who had tried either tobacco or cannabis (*r* = 0.74), we used grade instead of age as a control covariate, as it is more granular in this publicly available dataset.

We examined the potential mediating role of age at first use of cannabis or tobacco between the exogenous covariates and use/co-use outcomes (past 30-day and lifetime) using path analytic modeling. Two models were run, one for lifetime use and one for past 30-day use. The models examined both the direct (indicator variables covarying with use) and indirect effects (indicator variables covarying with use, through the mechanism of age at first use [mediator]) of the exogenous covariates on the use outcomes. We used Montecarlo integration to estimate indirect effects. Confidence intervals for these effects were used to determine statistical significance. For all models, the alpha level for statistical significance was set at 0.05.

## Results

### Descriptive results

Table 2 shows the descriptive statistics for the full sample and subsamples of youth who have used tobacco or cannabis in their lifetime and the past 30-days. In the full sample, weighted to the US population, 71% had never used tobacco or cannabis in their lifetime and 88% had not used in the past 30 days, 15% (lifetime) and 6% (past 30 day) had only used tobacco, 2% (lifetime) and 3% (past 30 day) had only used cannabis [the higher percentage for the past 30-day group is likely due to the small sample of youth who only have used cannabis in the past 30 days], and 12% (lifetime) and 3% (past 30 day) had used both at some time in their life. Across the board, youth factors of internalizing and externalizing problems, and sensation seeking were highest for youth who had used in the past 30 days (means: 2.74, 2.40, 7.66, respectively), followed by youth who had used tobacco or cannabis in their lifetime (means: 2.64, 2.27, 7.10, respectively) than the sample as a whole (means: 2.32, 2.00, 5.83, respectively). For youth who had used in the past 30 days, 64% began using one or both of these substances before the age of 15. For those who had used in their lifetime, but not necessarily in the past 30 days, this dropped to 56%. The proportion of single substance users (as compared to co-users) was higher in the past 30-day use group than the lifetime use group (71.74% and 58.12%, respectively).

**Table 2:** Lifetime, past 30-day, and full youth sample (including non-users) demographics, psychological constructs (sensation-seeking, internalizing, and externalizing), age at first use, and tobacco and cannabis use and co-use prevalence

	Lifetime users (n = 3857) M(SE)/frequency (%)	Past 30-day users (n = 1201) M(SE)/frequency (%)	Full youth sample (N = 13,651) M(SE)/frequency (%)
**Grade**	5.01 (0.03)	4.54 (0.04)	4.23 (0.10)
**Female gender**^a^	1174 (45.68%)	570 (47.30%)	6641 (48.70%)
**Hispanic**^a^	1117 (23.62%)	310 (20.65%)	3880 (22.46%)
**Race**^a^			
White	2519 (71.06%)	828 (74.95%)	8824 (70.02%)
Black	523 (14.69%)	143 (12.43%)	2056 (15.71%)
Other	633 (14.25%)	183 (12.62%)	2015 (14.27%)
**Internalizing** (range 1–4)	2.64 (0.02)	2.74 (0.03)	2.32 (0.01)
**Externalizing** (range 1–4)	2.27 (0.02)	2.40 (0.02)	2.00 (0.01)
**Sensation seeking** (range 1–15)	7.10 (0.05)	7.66 (0.08)	5.83 (0.03)
**Age at first use**^a^			
< 12	361 (10.93%)	95 (9.79%)	363 (10.18%)
12–14	1444 (44.73%)	505 (54.42%)	1518 (43.52%)
15–17	1341 (44.34%)	303 (35.80%)	1519 (46.30%)
Missing	711 (18.43%)	298 (24.81%)	n/a
**Single substance use**^a^	2246 (58.12%)	862 (71.74%)	n/a
Cannabis use	212 (5.38%)	287 (23.07%)	n/a
Tobacco use	2034 (52.74%)	575 (48.67%)	n/a
**Co-use**^a^	1611 (41.88%)	339 (28.26%)	n/a

Data are from the Population Assessment of Tobacco Health (PATH) study. ^a^Indicates frequency (%). Sample sizes are unweighted. Percentages (%) and standard errors (SE) are weighted to represent the US youth population (N =24,791,293). Missing data for predictors and covariates ranged from 0.08–4.72% (unweighted)

### Lifetime use model

**Fig. 1:**
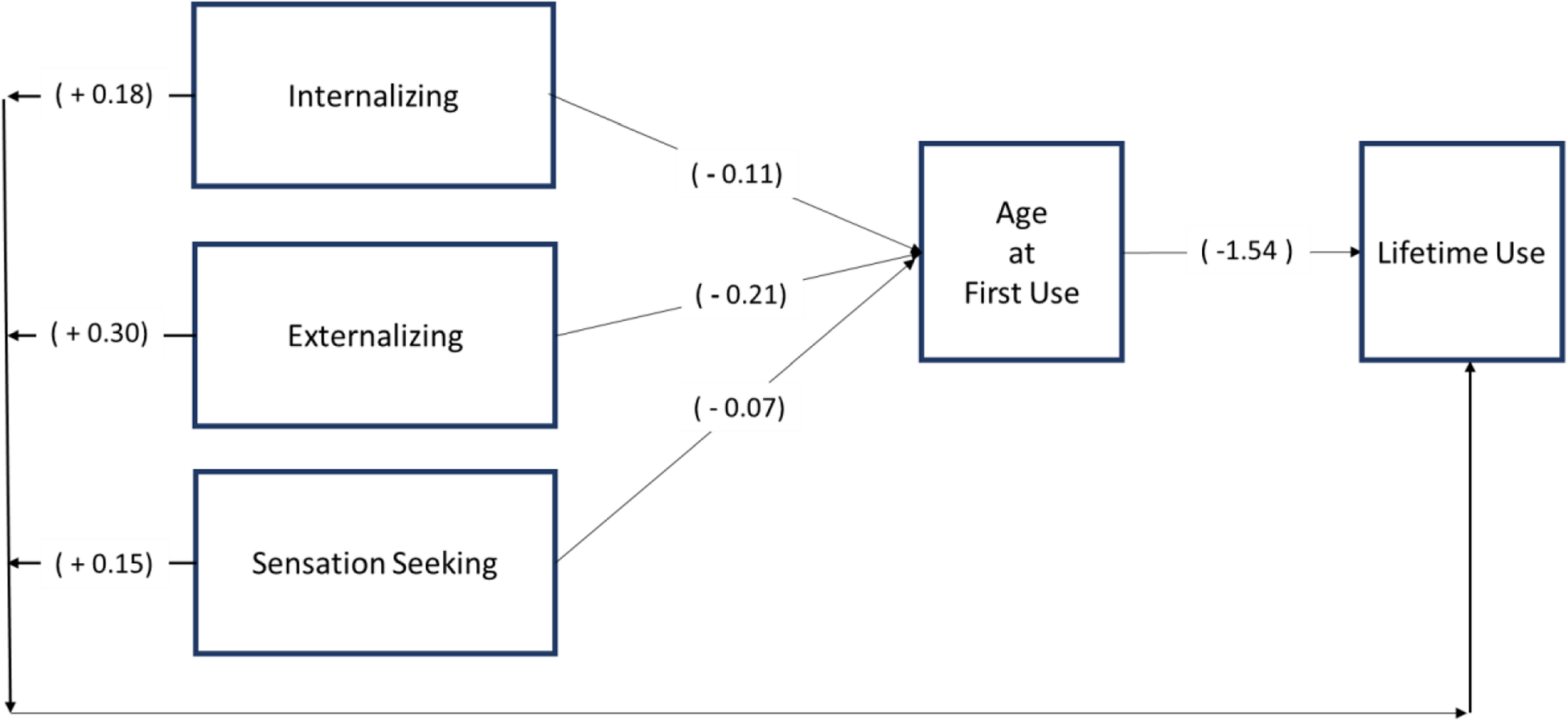
Significant indirect effects are also present for Internalizing (*B =* 0.18), Externalizing (*B =* 0.33) and sensation seeking (*B =* 0.10), *p* < 0.05. Estimates are in parentheses. Relationships between youth factors and lifetime tobacco and cannabis use mediated by age at first use of tobacco or cannabis

Table 3 shows the direct and indirect effects of each of the wave one youth factors on lifetime use status at wave two. Figure 1 gives an overview and visual depiction of these effects. Each of the exogenous covariates examined had a significant direct effect on youth’s propensity to co-use, as compared to using only tobacco or cannabis. Higher levels of internalizing, externalizing, and sensation seeking all increased the odds that a youth would use both tobacco and cannabis during their lifetime, with a one unit increase in internalizing indicating a 20% increase, externalizing indicating a 35% increase, and sensation seeking indicating a 16% increase in the odds of co-use (*p* < 0.05; OR 1.20, CI 0.12–0.24; OR 1.35, CI 0.20–0.40; OR 1.16, CI 0.13–0.17, respectively). In addition, each one unit increase in age at first use of tobacco or cannabis (e.g., “under age 12” to “age 12–14” or “age 12–14” to “15–17 years”), decreased the odds of the youth being a co-user, compared to being a single substance user, by 79% (*p* < 0.05, OR 0. 21, CI − 1.71 to − 1.37). The control factors of grade, gender, and Hispanic origin were significantly related to lifetime use status. Grade, gender, and race were significantly related to age at first use (see Table 3).

**Table 3:** Direct and specific indirect effects of youth demographics, psychological constructs of sensation-seeking, internalizing, and externalizing, and age at first use on lifetime tobacco and cannabis use status

Variables	Direct effect B (SE)	95% CI	OR	95% CI
**Co-use (as compared to single substance use)**				
→ Age at first use	− 1.54 (0.09)*	− 1.71, − 1.37	0.21	0.18, 0.25
→ Internalizing	0.18 (0.03)*	0.12, 0.24	1.20	1.12, 1.27
→ Externalizing	0.30 (0.05)*	0.20, 0.40	1.35	1.22, 1.50
→ Sensation seeking	0.15 (0.01)*	0.13, 0.17	1.16	1.14, 1.19
→ Grade	0.66 (0.02)*	0.62, 0.71	1.94	1.85, 2.03
→ Sex	− 0.17 (0.06)*	− 0.29, − 0.06	0.84	0.75, 0.94
→ Hispanic	− 0.27 (0.07)*	− 0.40, − 0.13	0.77	0.67, 0.88
→ Race	− 0.04 (0.04)	− 0.11, 0.03	0.96	0.89, 1.03
**Age at first use**				
→ Internalizing	− 0.11 (0.04)*	− 0.20, − 0.03	0.89	0.82, 0.97
→ Externalizing	− 0.21 (0.05)*	− 0.31, − 0.11	0.81	0.73, 0.90
→ Sensation seeking	− 0.07 (0.02)*	− 0.10, − 0.03	0.94	0.91, 0.97
→ Grade	0.58 (0.03)*	0.52, 0.64	1.78	1.68, 1.89
→ Sex	0.32 (0.07)*	0.18, 0.45	1.37	1.20, 1.57
→ Hispanic	0.13 (0.08)	− 0.03, 0.28	1.14	0.97, 1.32
→ Race	− 0.10 (0.04)*	− 0.18, − 0.02	0.91	0.84, 0.98
**Through age at first use**	**Indirect effect B (SE)**	**95% CI**		
→ Internalizing	0.17 (0.07)*	0.05, 0.30	–	–
→ Externalizing	0.33 (0.08)*	0.16, 0.49	–	–
→ Sensation seeking	0.10 (0.03)*	0.05, 0.15	–	–

Data are from the Population Assessment of Tobacco Health (PATH) study. *p < 0.05

Internalizing, externalizing, and sensation seeking were each associated with the age at which youth first used tobacco or cannabis. For each one unit increase in a youth’s rating of their own internalizing, the odds of being in the next older age at first use category decreased by 11% (*p* < 0.05, OR 0.89; CI − 0.20 to − 0.03). For each one unit increase in a youth’s rating of their own externalizing, the odds of being in the next older age at first use category decreased by 19% (*p* < 0.05, OR 0.81; CI − 0.31 to − 0.11). For each increase in a youth’s rating of their own sensation seeking, the odds of being in the next older age at first use category decreased by 6% (*p* < 0.05, OR 0.93; CI − 0.10 to − 0.03). In other words, youth who rated themselves higher on internalizing, externalizing, and sensation seeking had higher odds of being co-users, and also had higher odds of first using tobacco or cannabis at a younger age than the youth who rated themselves lower on these risk factors.

The indirect effects of internalizing, externalizing, and sensation seeking (*B =* 0.18, *SE =* 0.07; *B =* 0.33, *SE =* 0.08; *B =* 0.10, *SE =* 0.03, respectively, *p* < 0.05), through age at first use of tobacco or cannabis, on lifetime use status were each significant. These mediation results suggest that the association between these youth factors and tobacco and cannabis co-use can be partially explained by age of first use of these substances. That is, youth with higher levels of internalizing, externalizing, and sensation seeking are more likely to use these substances at an earlier age, which in turn is related to increased likelihood of lifetime co-use.

### Thirty-day use model

**Fig. 2:**
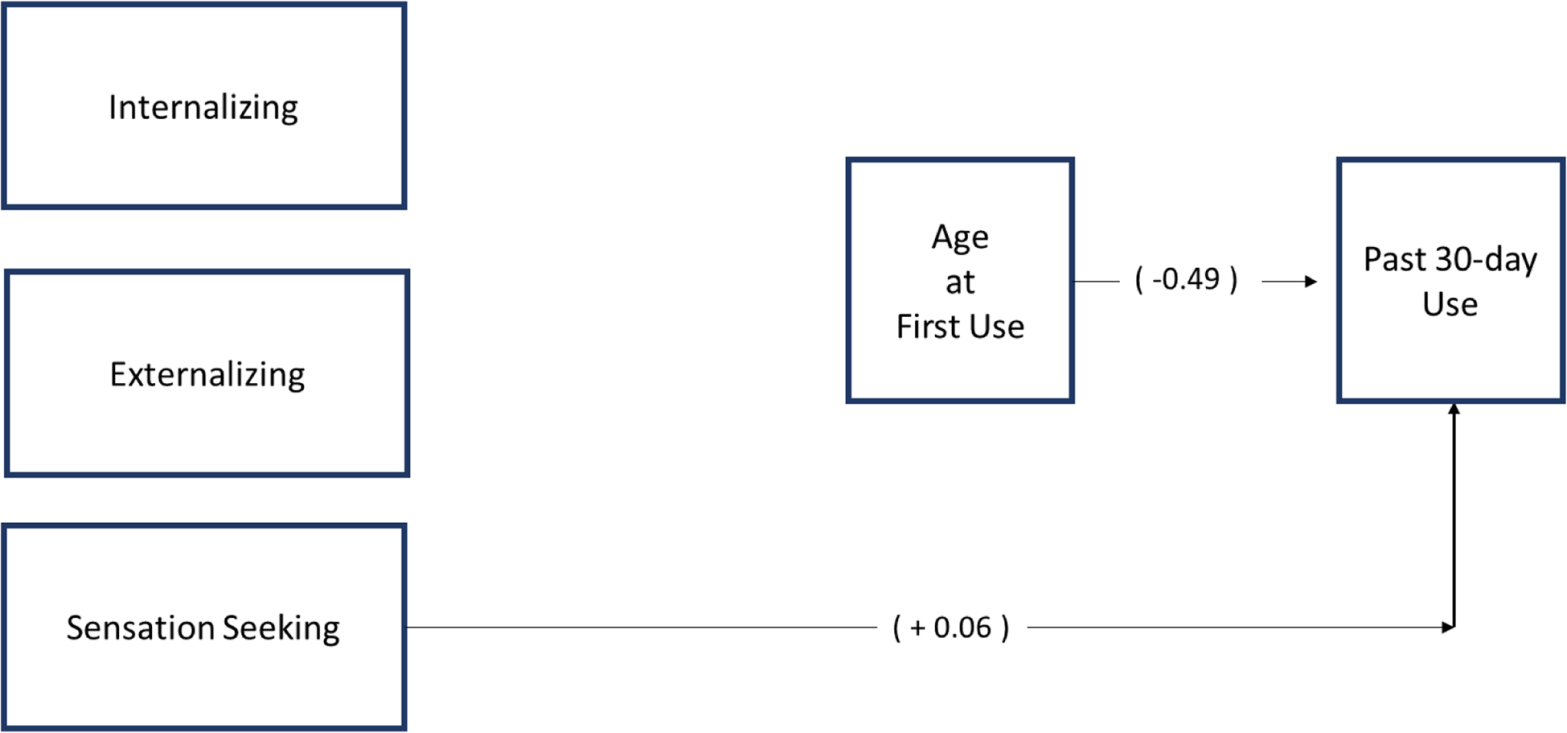
No indirect effects were significant (*p* > 0.05). Estimates are in parentheses. Relationships between youth factors and past 30-day tobacco and cannabis use mediated by age at first use of tobacco or cannabis

Table 4 shows the direct and indirect effects of each of the wave one youth factors on 30-days use status at wave two. Figure 2 gives an overview and visual depiction of these effects. Neither internalizing nor externalizing had a significant direct effect on the odds of co-use, as compared to single substance use, one year later. However, for each one unit increase in youth sensation seeking at wave one, the odds of co-use at wave two increase by 6% (OR 01.06; CI 0.01–0.12, *p* < 0.05). The age at which a youth first used tobacco or cannabis was also directly associated with the probability that they co-use the two substances. Each one unit increase in age at first use decreased the odds of youth co-use by 38% (OR 0.62; CI − 0.78 to − 0.20, *p* < 0.05).

**Table 4:** Direct and specific indirect effects of youth demographics, psychological constructs of sensation-seeking, internalizing, and externalizing, and age at first use on 30-day tobacco and cannabis use status

Variables	Direct effect B (SE)	95% CI	OR	95% CI
**Co-use (as compared to single substance use)**				
→ Age at first use	− 0.49 (0.15)*	− 1.78, − 0.20	0.62	0.46, 0.82
→ Internalizing	0.01 (0.08)	− 0.14, 0.16	1.01	0.87, 1.18
→ Externalizing	0.11 (0.12)	− 0.12, 0.33	1.11	0.89, 1.40
→ Sensation seeking	0.06 (0.03)*	0.01, 0.12	1.06	1.01, 1.13
→ Grade	0.15 (0.07)*	0.01, 0.29	1.16	1.01, 1.34
→ Sex	0.23 (0.16)	− 0.08, 0.53	1.25	0.92, 1.71
→ Hispanic	− 0.11 (0.17)	− 0.44, 0.22	0.90	0.65, 1.25
→ Race	− 0.14 (0.09)	− 0.32, 0.05	0.87	0.73, 1.05
**Age at first use**				
→ Internalizing	− 0.09 (0.05)	− 0.19, 0.00	0.91	0.83, 1.00
→ Externalizing	− 0.11 (0.06)	− 0.23, 0.01	0.90	0.80, 1.01
→ Sensation seeking	− 0.02 (0.02)	− 0.05, 0.01	0.99	0.96, 1.01
→ Grade	0.67 (0.03)*	0.62, 0.73	1.96	1.86, 2.07
→ Sex	0.30 (0.08)*	0.15, 0.46	1.35	1.16, 1.58
→ Hispanic	0.03 (0.09)	− 0.14, 0.20	1.03	0.87, 1.22
→ Race	− 0.13 (0.05)*	− 0.22, − 0.03	0.88	0.80, 0.97
**Through age at first use**	**Indirect effect B (SE)**	**95% CI**		
→ Internalizing	0.04 (0.03)	− 0.01, 0.10	–	–
→ Externalizing	0.05 (0.03)	− 0.01, 0.12	–	–
→ Sensation seeking	0.01 (0.01)	− 0.01, 0.02	–	–

Data are from the Population Assessment of Tobacco Health (PATH) study. *p < 0.05

In addition, internalizing, externalizing, and sensation seeking did not have indirect effects through age at first use on 30-day use outcome status, indicating that mediation is not present in this model. In regard to the control factors, only grade was significantly related to both age at first use and use status (see Table 4).

## Discussion

To our knowledge, this is the first study to examine the mediating role of age at first use in the effects of youth internalizing, externalizing, and sensation seeking on tobacco and cannabis co-use. This is an important step in understanding the complex relationships between youth factors and substance use, particularly co-use of tobacco and cannabis. Based on the SDM and the empirical support for the effect of having a later age at first use as being less risky than a younger age at first use, age at first use should be situated as a mediator in the models (Catalano and Hawkins 1996; Catalano et al. 1996). In agreement with our hypothesis, our findings suggest that mediation of the relationships between youth factors and use outcomes, by age at first use, is present in the model examining lifetime use supporting the use of the SDM in prediction of youth substance use. Furthermore, the continued presence of direct effects of the youth factors (internalizing, externalizing, and sensation seeking) in the lifetime use model suggests that these youth factors are not only working through their effect on age at first use, but also are directly impacting the outcome. These results are largely consistent with prior research on predictors of polysubstance use as compared to single substance use (Martinotti et al. 2009) and research on youth internalizing, externalizing, and sensation seeking, and co-use of tobacco and cannabis (Agrawal et al. 2012; Agrawal et al. 2006; Ball et al. 1994; Brook et al. 2010; Chen and Jacobson 2012).

However, different from our initial prediction, direct and indirect effects of the youth factors of internalizing and externalizing were no longer present when we moved from the lifetime to past 30-day use models. Although the reasons for this remain unknown, it is worth noting that the sample for the past 30-day use model was considerably smaller than the sample for lifetime use (n=1201 and 3857, respectively, with 339 past 30-day co-users). Thus, the differences between the results of these two models may be due to an issue of statistical power. This is evidenced by a notable diminished precision in the confidence intervals of the past 30-day use results when compared to the results of the lifetime use model (on average 50% larger), and may be one of the reasons that additional paths between the youth factors and use outcomes were present in the lifetime use model but not in the past 30-day use model.

Another potential explanation for these differences is that the past 30-day model contains youth with more severe use patterns. In this model, youth have all used these substances in the last month, whereas youth in the lifetime use model had simply used at least once, some time in their life span. As with the distinction between single substance use and co-use as levels of substance use severity, lifetime use and past 30-day use may also be viewed as levels of use severity, with youth co-using in the past 30 days potentially being the “most severe” group out of the four examined in this set of analyses (lifetime single substance use, past 30-day single substance use, lifetime co-use, past 30-day co-use).

It is important to note however that, while relationships in the past 30-day use model differed from the lifetime use model, a significant positive direct effect of sensation seeking was also observed in the past 30-day use model. In agreement with previous youth studies (Agrawal et al. 2012; Agrawal et al. 2006), these results suggest that, compared to internalizing and externalizing behaviors, sensation seeking may play a more prominent role on past 30 days co-use of tobacco and cannabis compared to past 30 days use of either one of these substances.

Finally, consistent with previous studies, an inverse relationship was found between age at first use and co-use (this was found for both the lifetime use and past 30-day use outcomes) (Agrawal et al. 2006; Montgomery 2015). This suggests that delaying age at first use with novel, evidence-based prevention efforts could impact future co-use for youth who have only tried tobacco or cannabis in their lifetime and haven’t graduated to the more frequent use pattern (i.e., past 30-day use). It is important, however, to interpret this with some caution because although recency and frequency of use are highly correlated, it is possible that youth who endorsed using in the past 30 days were also trying these substances for the first time at that point.

### Strengths and limitations

This study is strengthened by its use of a large, nationally representative dataset. These results are novel in that they compare predictors of co-use to those of single substance use, a unique and important differentiation.

Some limitations to this study should also be noted. Measures of substance use were obtained from adolescent self-reports, which have been shown to have only fair validity (Williams and Nowatzki 2005). The PATH study, however, attempted to alleviate this potential bias by using Audio Computer-Assisted Self-Interviewing (Hyland et al. 2016). This study focused on the use of cannabis and tobacco only- a more comprehensive view of substance use risk and protective factors may be obtained with the inclusion of additional substances and combinations thereof. This sample also does not represent people who are incarcerated or institutionalized, subgroups of youth who are typically at higher risk of use of both tobacco and cannabis. As such, it remains unclear if these findings can be generalized to those subgroups of youth. In addition, although our model is grounded in theory and previous research, it is possible that respondents’ use impacted their externalizing, sensation seeking, or internalizing, rather than these youth factors impacting age at first use as modeled. It is also possible that the youth factors examined may impact the intensity of use of either substance individually or in combination and not only whether co-use occurred or not. Due to the limitation of this secondary dataset not including questions on quantity of marijuana use we were unable to examine this. Lastly, some may propose that this study is limited by the combination of age at first use of cannabis and tobacco and that we would want to examine differences in age at first use separately due to differences in outcomes for the two. Due to multicollinearity between the two variables (r > 0.80) this was not a reasonable option.

### Future directions

This study contributes to a growing and important set of literature providing information on the factors and processes influencing youth tobacco and cannabis co-use. Studies such as these can contribute to the development of novel prevention policies and programs, as well as provide improvements to existing policies and programs (Botvin & Griffin 2014; Sun et al. 2008). Future research should consider focusing on (1) identifying factors that could strengthen or weaken these relationships (e.g., moderation of these effects by family factors), (2) engaging in lengthier longitudinal research (with greater than 1-year gap between the predictive and outcome variables) to better understand the timing and specific influences of the relationships found in the current study and examine patterns of use over time, (3) conducting a Latent Profile Analysis (LPA) of these and other potentially predictive factors with a categorical outcome of being either a “lifetime user” or “past 30-day user” in order to identify which youth are predisposed to membership in the riskier past 30-day use group, and (4) promoting utilization of prevention programs or interventions that target these youth factors in an effort to decrease co-use and stave off age at first use.
